# Big Data: Astronomical or Genomical?

**DOI:** 10.1371/journal.pbio.1002195

**Published:** 2015-07-07

**Authors:** Zachary D. Stephens, Skylar Y. Lee, Faraz Faghri, Roy H. Campbell, Chengxiang Zhai, Miles J. Efron, Ravishankar Iyer, Michael C. Schatz, Saurabh Sinha, Gene E. Robinson

**Affiliations:** 1 Coordinated Science Laboratory and Department of Electrical and Computer Engineering, University of Illinois at Urbana-Champaign, Urbana, Illinois, United States of America; 2 Department of Computer Science, University of Illinois at Urbana-Champaign, Urbana, Illinois, United States of America; 3 Carl R. Woese Institute for Genomic Biology & Department of Computer Science, University of Illinois at Urbana-Champaign, Urbana, Illinois, United States of America; 4 School of Library and Information Science, University of Illinois at Urbana-Champaign, Urbana, Illinois, United States of America; 5 Simons Center for Quantitative Biology, Cold Spring Harbor Laboratory, Cold Spring Harbor, New York, United States of America; 6 Carl R. Woese Institute for Genomic Biology, Department of Entomology, and Neuroscience Program, University of Illinois at Urbana-Champaign, Urbana, Illinois, United States of America

## Abstract

Genomics is a Big Data science and is going to get much bigger, very soon, but it is not known whether the needs of genomics will exceed other Big Data domains. Projecting to the year 2025, we compared genomics with three other major generators of Big Data: astronomy, YouTube, and Twitter. Our estimates show that genomics is a “four-headed beast”—it is either on par with or the most demanding of the domains analyzed here in terms of data acquisition, storage, distribution, and analysis. We discuss aspects of new technologies that will need to be developed to rise up and meet the computational challenges that genomics poses for the near future. Now is the time for concerted, community-wide planning for the “genomical” challenges of the next decade.

## Abstract 2

This perspective considers the growth of genomics over the next ten years and assesses the computational needs that we will face relative to other "Big Data" activities such as astronomy, YouTube, and Twitter.

We compared genomics with three other major generators of Big Data: astronomy, YouTube, and Twitter. Astronomy has faced the challenges of Big Data for over 20 years and continues with ever-more ambitious studies of the universe. YouTube burst on the scene in 2005 and has sparked extraordinary worldwide interest in creating and sharing huge numbers of videos. Twitter, created in 2006, has become the poster child of the burgeoning movement in computational social science [6], with unprecedented opportunities for new insights by mining the enormous and ever-growing amount of textual data [7]. Particle physics also produces massive quantities of raw data, although the footprint is surprisingly limited since the vast majority of data are discarded soon after acquisition using the processing power that is coupled to the sensors [8]. Consequently, we do not include the domain in full detail here, although that model of rapid filtering and analysis will surely play an increasingly important role in genomics as the field matures.

To compare these four disparate domains, we considered the four components that comprise the “life cycle” of a dataset: acquisition, storage, distribution, and analysis (Table 1).

**Table 1:** Four domains of Big Data in 2025. In each of the four domains, the projected annual storage and computing needs are presented across the data lifecycle.

Data Phase	Astronomy	Twitter	YouTube	Genomics
**Acquisition**	25 zetta-bytes/year	0.5–15 billion tweets/year	500–900 million hours/year	1 zetta-bases/year
**Storage**	1 EB/year	1–17 PB/year	1–2 EB/year	2–40 EB/year
**Analysis**	In situ data reduction	Topic and sentiment mining	Limited requirements	Heterogeneous data and analysis
	Real-time processing	Metadata analysis		Variant calling, ~2 trillion central processing unit (CPU) hours
	Massive volumes			All-pairs genome alignments, ~10,000 trillion CPU hours
**Distribution**	Dedicated lines from antennae to server (600 TB/s)	Small units of distribution	Major component of modern user’s bandwidth (10 MB/s)	Many small (10 MB/s) and fewer massive (10 TB/s) data movement

## Data Acquisition

The four Big Data domains differ sharply in how data are acquired. Most astronomy data are acquired from a few highly centralized facilities [9]. By contrast, YouTube and Twitter acquire data in a highly distributed manner, but under a few standardized protocols. Astronomy, YouTube, and Twitter are expected to show continued dramatic growth in the volume of data to be acquired. For example, the Australian Square Kilometre Array Pathfinder (ASKAP) project currently acquires 7.5 terabytes/second of sample image data, a rate projected to increase 100-fold to 750 terabytes/second (~25 zettabytes per year) by 2025 [9,10]. YouTube currently has 300 hours of video being uploaded every minute, and this could grow to 1,000–1,700 hours per minute (1–2 exabytes of video data per year) by 2025 if we extrapolate from current trends (S1 Note). Today, Twitter generates 500 million tweets/day, each about 3 kilobytes including metadata (S2 Note). While this figure is beginning to plateau, a projected logarithmic growth rate would suggest a 2.4-fold growth by 2025, to 1.2 billion tweets per day, 1.36 petabytes/year. In short, data acquisition in these domains is expected to grow by up to two orders of magnitude in the next decade.

**Fig 1:**
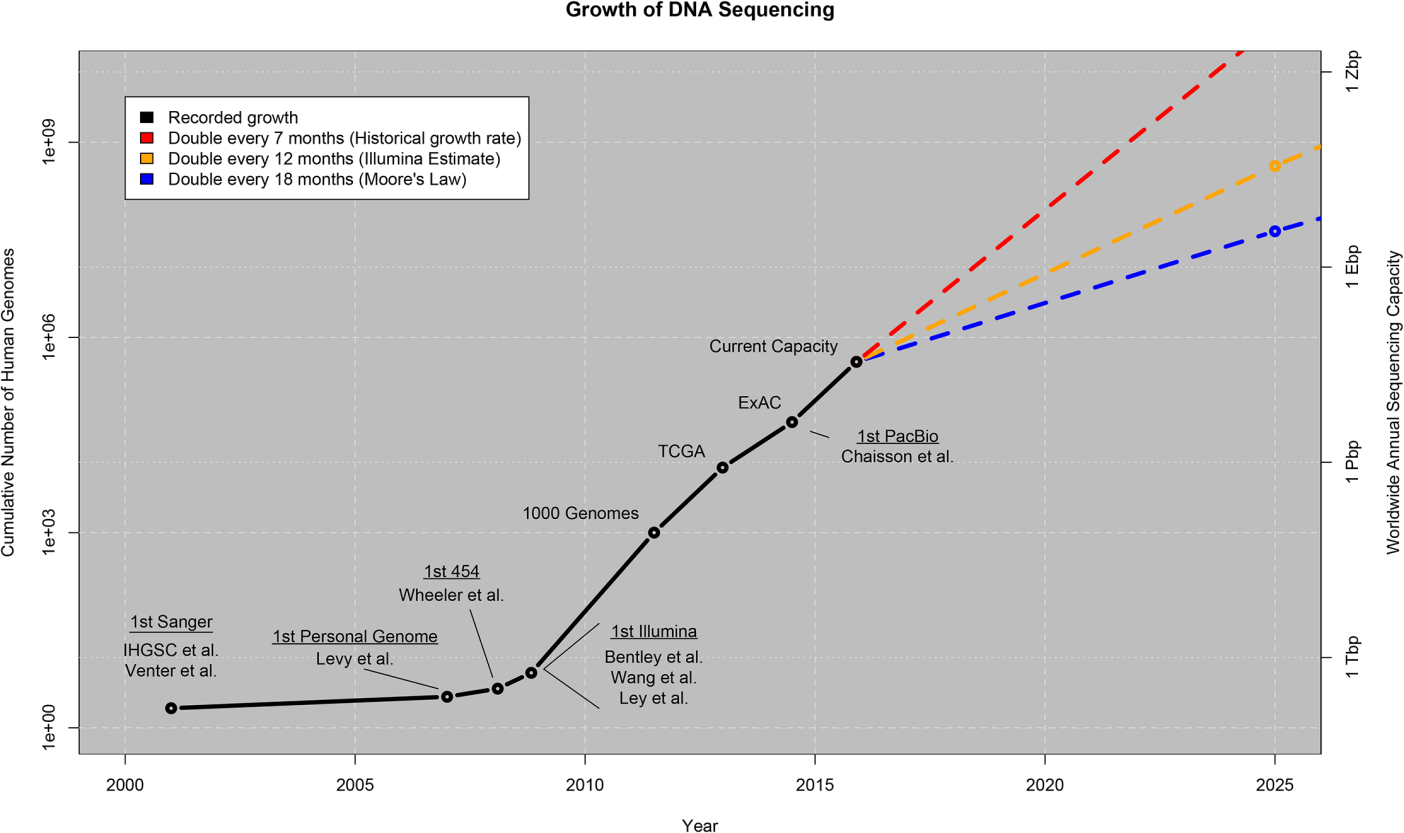
Growth of DNA sequencing. The plot shows the growth of DNA sequencing both in the total number of human genomes sequenced (left axis) as well as the worldwide annual sequencing capacity (right axis: Tera-basepairs (Tbp), Peta-basepairs (Pbp), Exa-basepairs (Ebp), Zetta-basepairs (Zbps)). The values through 2015 are based on the historical publication record, with selected milestones in sequencing (first Sanger through first PacBio human genome published) as well as three exemplar projects using large-scale sequencing: the 1000 Genomes Project, aggregating hundreds of human genomes by 2012 [3]; The Cancer Genome Atlas (TCGA), aggregating over several thousand tumor/normal genome pairs [4]; and the Exome Aggregation Consortium (ExAC), aggregating over 60,000 human exomes [5]. Many of the genomes sequenced to date have been whole exome rather than whole genome, but we expect the ratio to be increasingly favored towards whole genome in the future. The values beyond 2015 represent our projection under three possible growth curves as described in the main text.

For genomics, data acquisition is highly distributed and involves heterogeneous formats. The rate of growth over the last decade has also been truly astonishing, with the total amount of sequence data produced doubling approximately every seven months (Fig 1). The OmicsMaps catalog of all known sequencing instruments in the world [11] reports that currently there are more than 2,500 high-throughput instruments, manufactured by several different companies, located in nearly 1,000 sequencing centers in 55 countries in universities, hospitals, and other research laboratories. These centers range in size from small laboratories with a few instruments generating a few terabases per year to large dedicated facilities producing several petabases a year. (An approximate conversion factor to use in interpreting these numbers is 4 bases = 1 byte, though we will revisit this below.)

The raw sequencing reads used in most published studies are archived at either the Sequence Read Archive (SRA) maintained by the United States National Institutes of Health National Center for Biotechnology Information (NIH/NCBI) or one of the international counterparts. The SRA currently contains more than 3.6 petabases of raw sequence data (S1 Fig), which reflects the ~32,000 microbial genomes, ~5,000 plant and animal genomes, and ~250,000 individual human genomes that have been sequenced or are in progress thus far [12]. However, the 3.6 petabases represent a small fraction of the total produced; most of it is not yet in these archives. Based on manufacturer specifications of the instruments, we estimate the current worldwide sequencing capacity to exceed 35 petabases per year, including the sixteen Illumina X-Ten systems that have been sold so far [13], each with a capacity of ~2 petabases per year [14].

Over the next ten years, we expect sequencing capacities will continue to grow very rapidly, although the project growth becomes more unpredictable the further out we consider. If the growth continues at the current rate by doubling every seven months, then we should reach more than one exabase of sequence per year in the next five years and approach one zettabase of sequence per year by 2025 (Fig 1, Table 1). Interestingly, even at the more conservative estimates of doubling every 12 months (Illumina’s current own estimate [12]) or every 18 months (equivalent to Moore’s law), we should reach exabase-scale genomics well within the next decade. We anticipate this sequencing will encompass genome sequences for most of the approximately 1.2 million described species of plants and animals [15]. With these genomes, plus those of thousands of individuals of “high value” species for energy, environmental, and agricultural reasons, we estimate that there will be at least 2.5 million plant and animal genome sequences by 2025. For example, the genomics powerhouse BGI, in conjunction with the International Rice Research Institute and the Chinese Academy of Agricultural Sciences, has already sequenced 3,000 varieties of rice [16] and announced a massive project of their own to sequence one million plant and animal genomes [17]. The Smithsonian Institute also has similar plans to “capture and catalog all the DNA from the world’s flora and fauna.” There also will be genomes for several millions of microbes, with explosive growth projected for both medical and environmental microbe metagenomic sequencing [18,19].

These estimates, however, are dwarfed by the very reasonable possibility that a significant fraction of the world’s human population will have their genomes sequenced. The leading driver of this trend is the promise of genomic medicine to revolutionize the diagnosis and treatment of disease, with some countries contemplating sequencing large portions of their populations: both England [20] and Saudi Arabia [21] have announced plans to sequence 100,000 of their citizens, one-third of Iceland’s 320,000 citizens have donated blood for genetic testing [22], and researchers in both the US [23] and China [17] both aim to sequence 1 million genomes in the next few years. With the world’s population projected to top 8 billion by 2025, it is possible that as many as 25% of the population in developed nations and half of that in less-developed nations will have their genomes sequenced (comparable to the current worldwide distribution of Internet users [24]).

We therefore estimate between 100 million and as many as 2 billion human genomes could be sequenced by 2025, representing four to five orders of magnitude growth in ten years and far exceeding the growth for the three other Big Data domains. Indeed, this number could grow even larger, especially since new single-cell genome sequencing technologies are starting to reveal previously unimagined levels of variation, especially in cancers, necessitating sequencing the genomes of thousands of separate cells in a single tumor [10].

Moreover, the technology used to sequence DNA is deployed creatively for other applications (e.g., transcriptome, epigenome, proteome, metabolome, and microbiome sequencing) necessitating generating new sequencing data multiple times per person to monitor molecular activity [25]. These applications require precise quantitative counts of sequencing reads to capture diversity of expression or diversity of abundances, thus requiring millions of reads to accurately estimate underlying distributions as they change over time. For medicine, just having the genome will not be sufficient: for each individual, it will need to be coupled with other relevant ‘omics data sets, some collected periodically and from different tissues, to compare healthy and diseased states [26]. Computational challenges will increase because of dramatic increases in the total volume of genomic data per person, as will the complexities of integrating these diverse data sources to improve health and cure diseases. Genomics thus appears to pose the greatest challenges for data acquisition of the four Big Data domains.

## Data Storage

Data storage requirements for all four domains are projected to be enormous. Today, the largest astronomy data center devotes ~100 petabytes to storage, and the completion of the Square Kilometre Array (SKA) project is expected to lead to a storage demand of 1 exabyte per year. YouTube currently requires from 100 petabytes to 1 exabyte for storage and may be projected to require between 1 and 2 exabytes additional storage per year by 2025. Twitter’s storage needs today are estimated at 0.5 petabytes per year, which may increase to 1.5 petabytes in the next ten years. (Our estimates here ignore the “replication factor” that multiplies storage needs by ~4, for redundancy.) For genomics, we have determined more than 100 petabytes of storage are currently used by only 20 of the largest institutions (S1 Table).

Projections of storage requirements for sequence data depend on the accuracy and application of the sequencing. For every 3 billion bases of human genome sequence, 30-fold more data (~100 gigabases) must be collected because of errors in sequencing, base calling, and genome alignment. This means that as much as 2–40 exabytes of storage capacity will be needed by 2025 just for the human genomes (S3 Note). These needs can be diminished with effective data compression [27], but decompression times and fidelity are a major concern in compressive genomics [28].

Are the emerging “third-generation” single-molecule sequencing technologies with much longer reads, such as those from Pacific Biosciences and Oxford Nanopore, a computational panacea? Though error rates currently are higher and throughput lower than short-read technologies, as they mature, these technologies are starting to be used to sequence and assemble nearly entire chromosomes [29]. This will minimize the need to oversample as much, and eventually, the raw sequence data may not need to be stored at all. However, eliminating the need to store raw sequence data and only retaining complete genomes will have relatively little impact overall—perhaps one or two orders of magnitude less data storage. More significant reductions in storage demand will come when improvements in sequencing accuracy and database comprehensiveness reach the point at which genome sequences themselves do not need to be stored, just the list of variants relative to a reference collection (“delta encoding”) [30]. This works well for cataloging the simplest variants in a human genome, but it may not be as useful for complex samples, such as cancer genomes, that have many novel rearrangements and mutations. While certainly helpful, we thus do not expect long-read sequencing technology or delta encoding to solve the storage challenges for genome sequencing in 2025.

In contrast, we do see great opportunities for data reduction and real-time analysis of other ‘omics analysis. For example, once sequencing becomes fast enough and the methods mature enough to correctly infer transcript expression levels in real time, we anticipate that raw RNA-seq reads will no longer be stored, except for specific research purposes. Already several such “streaming” algorithms have been published for this purpose, performing as well as or superior to their nonstreaming counterparts [31]. For RNA-seq and other ‘omics applications, genomics will benefit greatly from the lessons learned in particle physics, in which in most cases raw data are discarded almost as fast as they are generated in favor of higher level and greatly compressed summaries.

Altogether, we anticipate the development of huge genomics archives used for storing millions of genomes along with the associated ‘omics measurements over time. Ideally, these archives will also collect or be linked to the patient phenotypic data, especially disease outcomes and treatments provided to support retrospective analysis as new relationships are discovered. To make it practical to search and query through such vast collections, the data will be stored in hierarchical systems that make data and their statistical summaries available at different levels of compression and latency, as used in astronomy [32] and text analysis [33]. Thus, although total genomic data could far exceed the demands for the others, with the right new innovations the net requirements could be similar to the domains of astronomy and YouTube.

## Data Distribution

Astronomy, YouTube, Twitter, and genomics also differ greatly in data distribution patterns. The major bandwidth requirement of the SKA project is to get data from its 3,000 antennae to a central server, requiring as much as 600 terabytes/second [34]. The bandwidth usage of YouTube is relatively small for a single download and well supported by the average consumer’s 10 Mbps connection, but aggregate needs worldwide are enormous, with estimates up to 240 petabytes/day (S4 Note) [35]. The distribution patterns of genomics data are much more heterogeneous, involving elements of both situations [36].

Genomic data are distributed in units spanning a wide range of sizes, from comparisons of a few bases or gene sequences to large multiterabyte bulk downloads from central repositories. For large-scale analysis, cloud computing is particularly suited to decreasing the bandwidth for distribution of genomic data [37] so that applications can run on remote machines that already have data [38]. Only small segments of code are uploaded and highly processed outputs are downloaded, thus significantly reducing the computing resources necessary for distribution.

But in addition to tailoring genomics applications for the cloud, new methods of data reliability and security are required to ensure privacy, much more so than for the other three domains. A serious breach of medically sensitive genomic data would have permanent consequences and could seriously hinder the development of genomic medicine. Homomorphic encryption systems, in which encrypted data can be analyzed and manipulated for certain controlled queries without disclosing the raw data, are currently too computationally expensive for widespread use, but these and related cryptographic techniques are promising areas of research [39].

## Data Analysis

Astronomy, YouTube, Twitter, and genomics differ most in computational requirements for data analysis. Astronomy data require extensive specialized analysis, but the bulk of this requirement is for in situ processing and reduction of data by computers located near the telescopes [40]. This initial analysis is daunting because of its real-time nature and huge data volumes but can often be effectively performed in parallel on thousands of cores. YouTube videos are primarily meant to be viewed, along with some automated analysis for advertisements or copyright infringements. Twitter data are the subject of intense research in the social sciences [41], especially for topic and sentiment mining, which is performed chiefly on textual “tweets” in the context of associated metadata (e.g., user demographics and temporal information).

Analysis of genomic data involves a more diverse range of approaches because of the variety of steps involved in reading a genome sequence and deriving useful information from it. For population and medical genomics, identifying the genomic variants in each individual genome is currently one of the most computationally complex phases. Variant calling on 2 billion genomes per year, with 100,000 CPUs in parallel, would require methods that process 2 genomes per CPU-hour, three-to-four orders of magnitude faster than current capabilities [42]. Whole genome alignment is another important form of genomic data analysis, used for a variety of goals, from phylogeny reconstruction to genome annotation via comparative methodologies. Just a single whole genome alignment between human and mouse consumes ~100 CPU hours [43]. Aligning all pairs of the ~2.5 million species expected to be available by 2025 amounts to 50–100 trillion such whole genome alignments, which would need to be six orders of magnitude faster than possible today.

Improvements to CPU capabilities, as anticipated by Moore’s Law, should help close the gap, but trends in computing power are often geared towards floating point operations and do not necessarily provide improvements in genome analysis, in which string operations and memory management often pose the most significant challenges. Moreover, the bigger bottleneck of Big Data analysis in the future may not be in CPU capabilities but in the input/output (I/O) hardware that shuttles data between storage and processors [44], a problem requiring research into new parallel I/O hardware and algorithms that can effectively utilize them.

## The Long Road Ahead

Genomics clearly poses some of the most severe computational challenges facing us in the next decade. Genomics is a “four-headed beast”; considering the computational demands across the lifecycle of a dataset—acquisition, storage, distribution, and analysis—genomics is either on par with or the most demanding of the Big Data domains. New integrative approaches need to be developed that take into account the challenges in all four aspects: it is unlikely that a single advance or technology will solve the genomics data problem. Several key technologies that are most critically needed to support future solutions are discussed in Box 1.

In human health, the major needs are driven by the realization that for precision medicine and similar efforts to be most effective, genomes and related ‘omics data need to be shared and compared in huge numbers. If we do not commit as a scientific community to sharing now, we run the risk of establishing thousands of isolated, private data collections, each too underpowered to allow subtle signals to be extracted. More than anything else, connecting these resources requires trust among institutions, scientists, and the public to ensure the collections will be used for medical purposes and not to discriminate or penalize individuals because of their genetic makeup.

Finally, the exascale data and computing centers that are emerging today to meet Big Data challenges in several domains (YouTube [50], Google [51], Facebook [51], and the National Security Agency [52]), are the result of far-sighted planning and commitment by the respective organizations. Now is the time for concerted, community-wide planning for the “genomical” challenges of the next decade.

**Box 1. Key Technological Needs for Big Data Genomics**

**(1) Acquisition**

The most important need to sustain the explosive growth in genomic data acquisition is continued advances in sequencing technologies to reduce costs, improve throughput, and achieve very high accuracy. The current costs of ~US$1,000 per human genome begin to make it practical to sequence human genomes in large numbers, especially for critical medical treatments, but to scale to populations of hundreds of millions to billions of genomes, costs must be reduced by at least another one to two orders of magnitude or more. For many medical applications, the time for sequencing must also be reduced so that it can be completed in near real time, especially to rapidly diagnosis acute infections and conditions. Finally, to make a genome sequence most useful, it must be paired with automated methods to collect metadata and phenotype data, all according to appropriate standards so that data collected in one environment can be compared to those collected in another.

**(2) Storage**

The community needs to start designing and constructing data centers with fast, tiered storage systems to query and aggregate over large collections of genomes and ‘omics data. There are new technologies on the horizon that will help support these needs, including 3-D memory, integrated computing technologies that overcome the I/O bottleneck, and networks that are two-to-five orders of magnitude faster because of optical switching [45,46]. Similarly, efficient compression and indexing systems are critical to make the best use out of each available byte while making the data highly accessible. We also expect algorithmic developments that can represent large collections of personal genomes as a compact graph, making it more efficient and robust to compare one genome to many others. Beyond these approaches, we see the rise of streaming approaches to make on-the-fly comparisons that will allow us to rapidly discard data, especially for sequencing applications that use the sequence data as a means to infer abundances or other molecular activity.

**(3) Distribution**

The most practical, and perhaps only, solution for distributing genome sequences at a population scale is to use cloud-computing systems that minimize data movement and maximize code federation [47]. New developments from companies such as Google, Amazon, and Facebook that include applications built to fit the frameworks of distributed computing efficient data centers and distributed storage and cloud computing paradigms will be part of the solution. Already, large cloud-based genomic resources are being developed using these technologies, especially to support the needs of the largest sequencing centers or to support the needs of large communities (BGI-cloud, TCGA, the International Cancer Genome Consortium [ICGC], etc.). To make these online systems most useful, the community needs to develop application programming interfaces (APIs) for discovering and querying large datasets on remote systems. The Global Alliance for Genomics and Health [48] and others are beginning to develop such standards for human genomic data, and we expect other communities to follow. Finally, authentication, encryption, and other security safeguards must be developed to ensure that genomic data remain private.

**(4) Analysis**

Our ultimate goal is to be able to interpret genomic sequences and answer how DNA mutations, expression changes, or other molecular measurements relate to disease, development, behavior, or evolution. Accomplishing this goal will clearly require integration of biological domain expertise, large-scale machine learning systems, and a computing infrastructure that can support flexible and dynamic queries to search for patterns over very large collections in very high dimensions. A number of “data science technologies,” including R, Mahout, and other machine learning systems powered by Hadoop and other highly scalable systems, are a start, but the current offerings are still difficult and expensive to use. The community would also benefit from libraries of highly optimized algorithms within a simple interface that can be combined and reused in many contexts as the problems emerge. Data science companies as well as open-source initiatives are already starting to develop such components, such as Amazon’s recent “Amazon Machine Learning” prediction system. But because genomics poses unique challenges in terms of data acquisition, distribution, storage, and especially analysis, waiting for innovations from outside our field is unlikely to be sufficient. We must face these challenges ourselves, starting with integrating data science into graduate, undergraduate, and high-school curricula to train the next generations of quantitative biologists, bioinformaticians, and computer scientists and engineers [49].

## Supporting Information

S1 Fig Growth of GenBank. The *y*-axis shows the total sequence in bp. (Blue = GenBank, red = whole genome shotgun [WGS] sequences.) Each line is double of the previous. The *x*-axis indicates time. Each line is 6 months after the previous. Source: http://www.ncbi.nlm.nih.gov/genbank/statistics.
(TIF)

S1 Note YouTube data estimates. (DOCX)

S2 Note Twitter data estimates. (DOCX)

S3 Note Human genomic data storage estimates for 2025. (DOCX)

S4 Note YouTube distribution statistics (current). (DOCX)

S1 Table Capacities of 20 major genomics institutions. The number of sequencers as listed from OmicsMaps.com and their storage capacities from the listed citation. These 20 institutions alone collectively have more than 100 PB of storage available.
(DOCX)

## Abbreviations

API: application programming interface
ASKAP: Australian Square Kilometre Array Pathfinder
CPU: central processing unit
ExAC: Exome Aggregation Consortium
ICGC: International Cancer Genome Consortium
I/O: input/output
NIH/NCBI: National Institutes of Health National Center for Biotechnology Information
SKA: Square Kilometre Array
SRA: Sequence Read Archive
TCGA: The Cancer Genome Atlas
WGS: whole genome shotgun

## References

[1] Mole, B. The gene sequencing future is here. 2014; http://www.sciencenews.org/article/gene-sequencing-future-here.

[2] Robinson G.E., et al, Creating a Buzz About Insect Genomes. Science, 2011 18: 1386. 10.1126/science.331.6023.1386 21415334

[3] Abecasis G.R., et al, An integrated map of genetic variation from 1,092 human genomes. Nature, 2012 491(7422): 56–65. 10.1038/nature11632 23128226 PMC3498066

[4] Chin L., Andersen J.N., and Futreal P.A., Cancer genomics: from discovery science to personalized medicine. Nature medicine, 2011 17(3): 297–303. 10.1038/nm.2323 21383744

[5] Exome Aggregation Consortium. Exome Aggregation Consortium ExAC Browser. 2015; http://exac.broadinstitute.org/.

[6] Giles J., Computational social science: Making the links. Nature, 2012 488(7412): 448–50. 10.1038/488448a 22914149

[7] Pak, A. and P. Paroubek, Twitter as a Corpus for Sentiment Analysis and Opinion Mining, in LREC. 2010. p. 1320–1326.

[8] O'Luanaigh, C. Animation shows LHC data processing. 2013; http://home.web.cern.ch/about/updates/2013/04/animation-shows-lhc-data-processing.

[9] Newman, R. and J. Tseng. Cloud Computing and the Square Kilometre Array. 2011; http://www.skatelescope.org/uploaded/8762_134_Memo_Newman.pdf.

[10] IBM Research. Square Kilometer Array: Ultimate Big Data Challenge. 2013; http://www.skatelescope.org/uploaded/8762_134_Memo_Newman.pdf.

[11] Omicsmap. Next Generation Genomics: World Map of High-throughput Sequencers. 2015; http://omicsmaps.com/.

[12] Regalado, A. EmTech: Illumina Says 228,000 Human Genomes Will Be Sequenced This Year. MIT Technology Review 2014 [cited 2015 April 28, 2015]; http://www.technologyreview.com/news/531091/emtech-illumina-says-228000-human-genomes-will-be-sequenced-this-year/.

[13] AllSeq. HiSeq X Ten. 2015 [cited 2015 April 1, 2015]; http://allseq.com/x-ten.

[14] Illumina. HiSeq X Series of Sequencing Systems. 2015 [cited April 28, 2015]; http://www.illumina.com/content/dam/illumina-marketing/documents/products/datasheets/datasheet-hiseq-x-ten.pdf.

[15] Mora C., et al, How Many Species Are There on Earth and in the Ocean? PLoS Biology, 2011; 9: e1001127 10.1371/journal.pbio.1001127 21886479 PMC3160336

[16] Li J.Y., Wang J., and Zeigler R.S., The 3,000 rice genomes project: new opportunities and challenges for future rice research. GigaScience, 2014 3: 8 10.1186/2047-217X-3-8 24872878 PMC4035671

[17] Zhu J., A year of great leaps in genome research. Genome medicine, 2012 4(1): 4 10.1186/gm303 22293069 PMC3334552

[18] Eisen J.A., Environmental shotgun sequencing: its potential and challenges for studying the hidden world of microbes. PLoS Biology, 2007 5(3): e82 17355177 10.1371/journal.pbio.0050082 PMC1821061

[19] Gevers D., et al, The Human Microbiome Project: a community resource for the healthy human microbiome. PLoS Biology, 2012 10(8): e1001377 10.1371/journal.pbio.1001377 22904687 PMC3419203

[20] Genomics England. The 100,000 Genomes Project. 2015; http://www.genomicsengland.co.uk/the-100000-genomes-project/.

[21] Briggs, H (2013) Hundred thousand genomes to be mapped in Saudi Arabia. BBC News. http://www.bbc.com/news/health-25216135

[22] Sulem P., et al, Identification of a large set of rare complete human knockouts. Nature genetics, 2015; 47: 448–452. 10.1038/ng.3243 25807282

[23] Kaiser, J. White House fleshes out Obama’s $215 million plan for precision medicine. Science Insider 2015; http://news.sciencemag.org/biology/2015/01/white-house-fleshes-out-obama-s-215-million-plan-precision-medicine.

[24] Internet World Stats. 2015; http://www.internetworldstats.com/stats.htm.

[25] Soon W.W., Hariharan M., and Snyder M.P., High-throughput sequencing for biology and medicine. Molecular systems biology, 2013 9: 640 10.1038/msb.2012.61 23340846 PMC3564260

[26] Chen R., et al, Personal omics profiling reveals dynamic molecular and medical phenotypes. Cell, 2012 148(6): 1293–307. 10.1016/j.cell.2012.02.009 22424236 PMC3341616

[27] Hsi-Yang Fritz M., et al, Efficient storage of high throughput DNA sequencing data using reference-based compression. Genome research, 2011 21(5): 734–40. 10.1101/gr.114819.110 21245279 PMC3083090

[28] Loh P.-R., Baym M., and Berger B., Compressive genomics. Nature Biotechnology, 2012 30: 627–630. 10.1038/nbt.2241 22781691

[29] Koren S. and Phillippy A.M., One chromosome, one contig: complete microbial genomes from long-read sequencing and assembly. Current opinion in microbiology, 2015 23C: 110–120. 10.1016/j.mib.2014.11.014 25461581

[30] Christley S., et al, Human genomes as email attachments. Bioinformatics, 2009 25(2): 274–5. 10.1093/bioinformatics/btn582 18996942

[31] Patro R., Mount S.M., and Kingsford C., Sailfish enables alignment-free isoform quantification from RNA-seq reads using lightweight algorithms. Nature biotechnology, 2014 32(5): 462–4. 10.1038/nbt.2862 24752080 PMC4077321

[32] Golden A., Djorgovski S., and Greally J., Astrogenomics: big data, old problems, old solutions? Genome Biology, 2013 8: 129. 10.1186/gb-2013-14-8-129 PMC4053852 23953643

[33] Djoerd Hiemstra C.H., Brute Force Information Retrieval Experiments using MapReduce'. ERCIM News, 2012 89: 31–32.

[34] Smith, B., Data Transport for the Square Kilometre Array, in UbuntuNet Alliance Annual Conference. 2012. 15–22.

[35] Global Internet Phenomena Report. 2013; http://www.sandvine.com/downloads/general/global-internet-phenomena/2013/2h-2013-global-internet-phenomena-report.pdf.

[36] Sboner A., et al, The real cost of sequencing: higher than you think! Genome Biology, 2011 12: 125 10.1186/gb-2011-12-8-125 21867570 PMC3245608

[37] Marx, V., Drilling into big cancer-genome data. Nature Methods, 2013. 10: p. 293–297. 10.1038/nmeth.2410 23538863

[38] Baker M., Next-generation sequencing: adjusting to data overload. Nature Methods, 2010 7: 495–499.

[39] Erlich Y. and Narayanan A., Routes for breaching and protecting genetic privacy. Nature reviews. Genetics, 2014 15(6): 409–21. 10.1038/nrg3723 24805122 PMC4151119

[40] Norris, R.P., Data Challenges for Next-generation Radio Telescopes. 2011.

[41] Kumar S., Morstatter F., and Liu H., Twitter Data Analytics 2014: Springer.

[42] Langmead B., et al, Searching for SNPs with cloud computing. Genome Biol, 2009 10(11): R134 10.1186/gb-2009-10-11-r134 19930550 PMC3091327

[43] Kurtz S., et al, Versatile and open software for comparing large genomes. Genome Biol, 2004 5(2): R12 14759262 10.1186/gb-2004-5-2-r12 PMC395750

[44] Trelles O., et al, Big data, but are we ready? Nature reviews. Genetics, 2011 12(3): 224. 10.1038/nrg2857-c1 21301471

[45] Loh, G.H. 3D-stacked memory architectures for multi-core processors. in ACM SIGARCH. 2008.

[46] Chan, V.W., et al. Optical flow switching: An end-to-end “UltraFlow” architecture. in 15th International Conference on Transparent Optical Networks (ICTON). 2013. IEEE. 10.1109/ICTON.2013.6602704

[47] Schatz M.C., Langmead B., and Salzberg S.L., Cloud computing and the DNA data race. Nature biotechnology, 2010 28(7): 691–3. 10.1038/nbt0710-691 20622843 PMC2904649

[48] Global Alliance for Genomics and Health. Global Alliance for Genomics and Health. 2015 April 28, 2015]; http://genomicsandhealth.org/.

[49] Schatz, M.C., Computational thinking in the era of big data biology. Genome Biology, 2012. 13(11): 177. 10.1186/gb-2012-13-11-177 PMC3580488 23194371

[50] Hollis, C. EMC's Record Breaking Product Launch. 2011 April 28, 2015]; http://chucksblog.emc.com/chucks_blog/2011/01/emcs-record-breaking-product-launch.html.

[51] Hoff, T. How Google Backs up the Internet along with Exabytes of other data. 2014; http://highscalability.com/blog/2014/2/3/how-google-backs-up-the-internet-along-with-exabytes-of-othe.html.

[52] Daily Kos. Utah Data Center stores data between 1 exabyte and 1 yottabyte. 2013; http://www.dailykos.com/story/2013/08/05/1228923/-Utah-Data-Center-stores-data-between-1-exabyte-and-1-yottabyte.

