# Supplementary figures and images for: Big Data: Astronomical or Genomical?
Source: PLoS Biol. 2015 Jul 7;13(7):e1002195. doi: 10.1371/journal.pbio.1002195 (PMC4494865; doi:10.1371/journal.pbio.1002195)

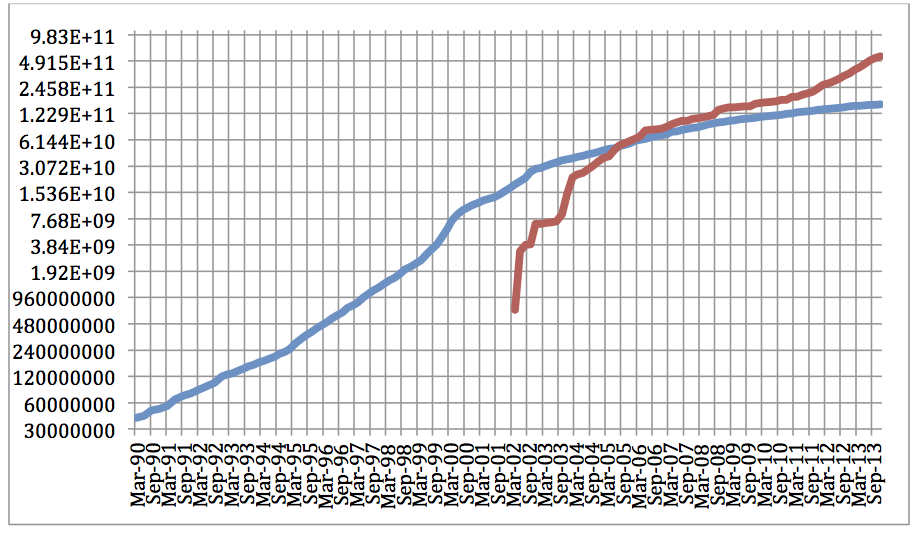

Supplement: S1 Fig — The y-axis shows the total sequence in bp. (Blue = GenBank, red = whole genome shotgun [WGS] sequences.) Each line is double of the previous. The x-axis indicates time. Each line is 6 months after the previous. Source: http://www.ncbi.nlm.nih.gov/genbank/statistics. (TIF) [file pbio.1002195.s001.tif]
