# Supplementary material for: Big Data: Astronomical or Genomical?
Source: PLoS Biol. 2015 Jul 7;13(7):e1002195. doi: 10.1371/journal.pbio.1002195 (PMC4494865; doi:10.1371/journal.pbio.1002195)
Supplement: S1 Note — (DOCX) [file pbio.1002195.s002.docx]

The most reliable reference for estimations is the official YouTube statistics [1]

- 2015: 300 hours of video are uploaded to YouTube every minute [1]

- mid 2012: 72 hours [2]

- early 2012: 60 hours [3]

- 2011: 48 hours [4]

- 2010: 35 hours [5]

- 2009: 20 hours [6]

- 2007: 10 hours [5,6]

Using the above numbers, we may estimate a growth rate in the data acquisition rate (hours of video uploaded per minute), and thus a projected acquisition rate for 2025, as shown in the following table.

| Year | Video hours uploaded every minute | Growth/yr in hr/min upload |
| --- | --- | --- |
| 2005 | 0 |  |
| 2007 | 10 | 5 |
| 2009 | 20 | 5 |
| 2010 | 35 | 15 |
| 2011 | 48 | 13 |
| 2012 | 72 | 24 |
| 2015 | 300 | 76 |
| 2025 (estimate, linear) | 1060 |  |
| 2025 (estimate, quadr) | 1,640 |  |

The above data acquisition rates can be used to estimate a cumulative value on the video data as shown in the following table. Note: Conversion from video hours to size in bytes is done as 1 hour ≈ 2.4 GB. We project to upload volumes in 2025 based on the change from 2012 to 2015, assuming linear growth and quadratic growth respectively.

| Year | Hr every min | Trapezoid area (525949 mins/year) | Cumsum (total hrs uploaded by date) | Cumulative YouTube size (GB, 1hr~2.4GB) | Cumulative YouTube size (PB) | Growth/yr in Cumul size (PB/yr) | Annual Upload volume (PB/yr) |
| --- | --- | --- | --- | --- | --- | --- | --- |
| 2005 | 0 | 0 | 0 | 0 | 0.00 |  |  |
| 2007 | 10 | 5259490 | 5259490 | 12622776 | 12.62 | 6.31 | 12.62 |
| 2009 | 20 | 15778470 | 21037960 | 50491104 | 50.49 | 18.93 | 25.25 |
| 2010 | 35 | 14463597.5 | 35501557.5 | 85203738 | 85.20 | 34.71 | 44.18 |
| 2011 | 48 | 21826883.5 | 57328441 | 137588258.4 | 137.59 | 52.38 | 60.59 |
| 2012 | 72 | 31556940 | 88885381 | 213324914.4 | 213.32 | 75.74 | 90.88 |
| 2015 | 300 | 293479542 | 382364923 | 917675815.2 | 917.68 | 234.78 | 378.68 |
| 2025 (est, linear) | 1060 |  |  |  | 3265.51 |  | 1338.01 |
| 2025 (est, quadr.) | 1640 |  |  |  |  |  | 2070.14 |

**Supplemental References**

[1]  <http://www.youtube.com/yt/press/statistics.html>

[2]  <http://paulwallbank.com/2012/08/23/how-much-server-space-do-internet-companies-need-to-run-their-sites/>

[3]  <https://sumanrs.wordpress.com/2012/04/14/youtube-yearly-costs-for-storagenetworking-estimate/>

[4]  <http://searchenginewatch.com/sew/study/2073962/youtube-statistics-48-hours-video-uploaded-minute-billion-views-day>

[5]  <http://www.reelseo.com/youtube-35-hours-minute/>

[6]  <http://youtube-global.blogspot.com/2009/05/zoinks-20-hours-of-video-uploaded-every_20.html>
