# Supplementary material for: Big Data: Astronomical or Genomical?
Source: PLoS Biol. 2015 Jul 7;13(7):e1002195. doi: 10.1371/journal.pbio.1002195 (PMC4494865; doi:10.1371/journal.pbio.1002195)
Supplement: S2 Note — (DOCX) [file pbio.1002195.s003.docx]

Our estimates of Twitter data volume are based on personal communications with Qiaozhu Mei and from the following reference: <http://www.internetlivestats.com/twitter-statistics/>

The size of a single tweet is 140 characters, i.e., ~200 bytes, and with meta-data this comes to about 3 KB on average, excluding images.

The growth of number of tweets and cumulative size can be estimated from the above as in the following table.

| Year | Tweets per day | Cumulative Twitter size (GB) (1 tweet = 3 KB) | Annual upload volume (GB/yr) |
| --- | --- | --- | --- |
| 2006 | 1 | 0.00 |  |
| 2007 | 5,000 | 2.74 | 2.74 |
| 2008 | 300,000 | 169.73 | 166.99 |
| 2009 | 2,500,000 | 1702.73 | 1533.00 |
| 2010 | 50,000,000 | 30446.48 | 28743.75 |
| 2011 | 250,000,000 | 194696.48 | 164250.00 |
| 2012 | 340,000,000 | 517721.48 | 323025.00 |
| 2013 | 500,000,000 | 977621.48 | 459900.00 |
| 2014 | 500,000,000 | 1525121.48 | 547500.00 |
| 2015 | 500,000,000 | 2072621.48 | 547500.00 |

The estimated current annual upload volume is 547 terabytes (difference between last two rows above). While the statistics from the last three years suggests a plateauing of the number of tweets, we used a logarithmic fit to the “tweets per day” statistic from 2006 onwards, to estimate the number of tweets in 2025 at 1,239,840,000 per day (~2.4x growth over 2015), corresponding to an annual upload volume of 1.36 petabytes per year.
