# Supplementary material for: Big Data: Astronomical or Genomical?
Source: PLoS Biol. 2015 Jul 7;13(7):e1002195. doi: 10.1371/journal.pbio.1002195 (PMC4494865; doi:10.1371/journal.pbio.1002195)
Supplement: S3 Note — (DOCX) [file pbio.1002195.s004.docx]

One human genome is 3 billion bp

100 million human genomes = 10^8 x 3 x 10^9 bp = 300 petabases.

2 billion human genomes = 2 x 10^9 x 3 x 10^9 bp = 6 exabases.

Because of errors in sequencing, base calling and genome alignment, ~30-fold more data is collected, so the raw data amounts to 9 exabases to 150 exabases.

At 4 bases per byte (uncompressed), this gives us a range of ~2 EB – 40 EB.
