# Supplementary material for: Big Data: Astronomical or Genomical?
Source: PLoS Biol. 2015 Jul 7;13(7):e1002195. doi: 10.1371/journal.pbio.1002195 (PMC4494865; doi:10.1371/journal.pbio.1002195)
Supplement: S4 Note — (DOCX) [file pbio.1002195.s005.docx]

According to YouTube statistics (<https://www.youtube.com/yt/press/statistics.html>), hundreds of millions of hours of video are watched every day.

Using the conversion factor of 1 hour video = 2.4 GB, we have 100 million hours = 240 PB of video data downloaded per day.
