# Supplementary material for: Big Data: Astronomical or Genomical?
Source: PLoS Biol. 2015 Jul 7;13(7):e1002195. doi: 10.1371/journal.pbio.1002195 (PMC4494865; doi:10.1371/journal.pbio.1002195)
Supplement: S1 Table — The number of sequencers as listed from OmicsMaps.com and their storage capacities from the listed citation. These 20 institutions alone collectively have more than 100 PB of storage available. (DOCX) [file pbio.1002195.s006.docx]

| Institute | Num. of Sequencers (omicsmap.com) | Storage  (PB) | Citation |
| --- | --- | --- | --- |
| BGI (formerly Beijing Genomics Institute) | 166 | 9 | [1] |
| Broad Institute | 101 | 10 | [2] |
| The Genome Center at Washington University | 38 | 10 | [3] |
| Wellcome Trust Sanger Institute | 38 | 22 | [4] |
| Human Genome Sequencing Centre, Baylor College of Medicine | 32 | 3.2 | [5] |
| Macrogen | 27 | 4 | [6] |
| NY Genome Center | 27 | 1 | [7] |
| McGill University and Génome Québec Innovation Centre | 22 | 5 | [8] |
| Yale Center for Genome Analysis | 20 | 2 | [9] |
| DOE Joint Genome Institute | 15 | 2 | [10] |
| Beijing Institute of Genomics | 15 | 1 | [11] |
| CSHL | 15 | 3 | [12] |
| Ontario Institute for Cancer Research | 14 | 3.5 | [13] |
| Canada's Michael Smith Genome Sciences Centre | 13 | 7 | [14] |
| Centro Nacional de Análisis Genómico (CNAG) | 12 | 2 | [15] |
| UCSF | 2 | 7 | [16] |
| St Jude | 8 | 2 | [17] |
| UCSC / CGHUB | 6 | 5 | [18] |
| UMD-IGS | 5 | 1 | [19] |
| EBI | 0 | 1.2 | [20] |
|  |  |  |  |
| *Sum* | ***576*** | ***100.9*** |  |

**Storage Citations**

| [1] | <http://www.emc.com/collateral/customer-profiles/h12077-cp-bgi-shenzen.pdf> |
| --- | --- |
| [2] | <http://www.broadinstitute.org/blog/five-questions-martin-leach> |
| [3] | <http://www.stltoday.com/lifestyles/health-med-fit/medical/genome-institute-at-washington-u-works-on-project-to-help/article_10d9e368-43d6-5a8e-b21a-516bdc14bc53.html> |
| [4] | <http://insidehpc.com/2013/10/07/sanger-institute-deploys-22-petabytes-lustre-powered-ddn-storage/> |
| [5] | <http://www.businesscloudnews.com/2013/10/28/baylor-college-of-medicine-shifts-human-genome-sequencing-project-to-public-cloud/> |
| [6] | <http://www.macrogen.com/eng/business/bio_overview.html> |
| [7] | <http://www.nygenome.org/jobs/data-architect-2/> |
| [8] | <http://www.hpc.mcgill.ca/index.php/services> |
| [9] | <http://www.genomeweb.com/sequencing/qa-shrikant-mane-sequencing-yale-center-genome-analysis> |
| [10] | <http://www.thefreelibrary.com/Joint+Genome+Institute+Fuels+Clean+Energy+Pursuits+With+Isilon...-a0227853222> |
| [11] | <http://connection.ebscohost.com/c/articles/50843877/beijing-genomics-institute-deploys-1-petabyte-storage-dna-sequencing> |
| [12] | <http://schatzlab.cshl.edu/apply/> |
| [13] | <http://www.recruitingsite.com/csbsites/oicr/JobDescription.asp?JobNumber=716250> |
| [14] | <http://openparliament.ca/committees/health/41-1/68/dr-marco-marra-1/only/> |
| [15] | <http://www.inab.org/job-opportunities/post-doctoral-researcher-and-technician-in-functional-bioinformatics/> |
| [16] | <http://www.hpcwire.com/2011/01/07/the_ucsf_institute_for_human_genetics_analyzes_7_petabytes_of_data_with_help_from_dell/> |
| [17] | <http://www.curechildhoodcancer.org/wp-content/uploads/2010/06/St.-Jude-grant-release-03.08.12.pdf> |
| [18] | <http://news.sciencemag.org/2012/05/worlds-largest-hub-cancer-genomes-opens> |
| [19] | <http://www.igs.umaryland.edu/resources/irc/it.php> |
| [20] | <http://geant2.archive.geant.net/upload/pdf/Cameron.pdf> |
